# Transition to Adult Care for Young People Living with HIV

**DOI:** 10.1007/s11904-025-00730-7

**Published:** 2025-03-01

**Authors:** Hannah Chew, Neerav Desai

**Affiliations:** 1 Vanderbilt University School of Medicine, Nashville, TN USA; 2 Department of Pediatrics, Vanderbilt University Medical Center, 719 Thompson Lane, Suite 36300, Nashville, TN 37204 USA

**Keywords:** Transition to adult care, Young people living with HIV, HIV care transfer

## Abstract

**Purpose of Review:**

The purpose of this narrative review is to delineate the challenges of transitioning young people living with HIV (YPLHIV) to adult-based care and to review recent literature including both qualitative and interventional studies focused on the process of transitioning.

**Methods:**

A search in PubMed and Embase was conducted using the key words “adolescent.” “young adult,” “transition to adult care,” “HIV," and “AIDS,” including only articles published from 2019 onwards. Conference proceedings from major peer-reviewed conferences focused on YPLHIV were manually searched for studies from January 2021 to December 2023. Data extraction included variables such as study type, participant ages, location, and, for intervention studies, detailed descriptions and outcomes, which were further categorized into themes. Results are included in Table 1 and Table 2.

**Recent Findings:**

Experts still debate about what a successful transition means which makes studying it harder. Challenges to successful transition include heterogeneity of the population, inconsistency with transition timing, mobility, and stigma. Recent qualitative studies that elicit feedback from stake holders reveal individual barriers such as lack of self-efficacy and fears of successive disclosures. A major facilitator to successful transition is having youth-friendly services in the adult clinic. Interventional studies emphasize the evidence for transition readiness assessments, transition protocols, mobile health engagement, transition clinics, and health care transition navigation.

**Summary:**

Health care teams who care for YPLHIV before, during, and after transition need to recognize how vulnerable this population can be. Therefore, transition must be formally addressed and grounded in the local settings and needs. Simple interventions have the potential to improve transition outcomes.

## Introduction

Transitioning young people living with HIV (YPLHIV) to adult-based care is a good problem to have. It represents a “problem” that has been created by the advent of highly active anti-retroviral therapy (ART) in the last 20 years, programs which target prevention of mother to child transmission, and efforts to improve diagnoses, linkage, engagement, and adherence among YPLHIV over the last several years. The American Academy of Pediatrics (AAP) defines health care transition as “the process of moving from a child to an adult model of healthcare” [1]. The Society for Adolescent Health and Medicine (SAHM), the AAP, and the World Health Organization (WHO) all recommend support and structure during transition to adult care for YPLHIV [2–4]. Despite these interventions and recommendations, many YPLHIV fail to transition to adult care without disruptions in treatment and increased rates of morbidity and mortality [5, 6]. This article reviews recent literature, both qualitative and interventional, that centers on transitioning to adult care for YPLHIV.

### What is a Successful Transition?

Researchers, policy makers, and health care providers still debate about what is meant by a successful transition to adult care for YPLHIV. Some groups define success to include successful linkage to adult care, appropriate retention in the adult care setting, and viral suppression after transition [7]. Other definitions include engagement with the adult clinic irrespective of viral suppression [8]. More broadly successful transition could also include self-management of health, which includes knowing how and when to make appointments, navigating health insurance, maintaining a structure that promotes adherence, and advocating for comprehensive health care needs. Globally, we want young adults to “live with HIV” which means they can pursue their educational, vocational, and social goals after their transition to adulthood.

### Why is Transition to Adult Care Difficult for YPLHIV?

There are several key drivers which make transitioning YPLHIV to adult care challenging. First, YPLHIV are a heterogenous population. Second, there is inconsistency with the timing and age of transition by location. Third, adolescence and young adulthood is a time of mobility. Fourth, YPLHIV are dealing with stigma and disclosure concerns.

### Heterogenous Population

Health care providers need to recognize that YPLHIV are a heterogenous population. Some YPLHIV have perinatal-acquired HIV (PAH) (traditionally termed vertical transmission). Other YPLHIV have non-perinatal acquired HIV (nPAH) (traditionally termed horizontal transmission) which could be through sexual contact, sexual assault, injection drug use, or contaminated needles and procedures. Each of these groups face challenges with the health care system especially around the time of transition to adult care [2, 7].

Young people with PAH face numerous challenges to transition to adult care. First and foremost, many of these individuals deal with familial trauma which could include death of parents or siblings due to HIV. Many young people with PAH face issues of displacement, orphanhood, fleeing violence, refugee camp living, and adoption all of which can affect physical and mental well-being. A recent study highlighted the diversity of youth with PAH living in the southeastern US including adoptees, refugees, and other immigrants from numerous cultural backgrounds [9]. Transition education and preparation must be culturally relevant and appropriate for these individuals and their families [10].

Young people with nPAH also face significant challenges with transition to adult care. Often these individuals are sexual and gender minorities and are the victim of discrimination and health inequalities. Common themes emerged in qualitative analyses of YPLHIV with nPAH including stigma, emotional coping with new HIV diagnosis, and mental health issues [11, 12]. Indeed, all YPLHIV are bringing lived experiences which could include trauma, stigma, and discrimination to the transition process.

### Inconsistency with Timing of Transition

There are very few agreed upon recommendations as to the timing of transition to adult care. Many groups can agree that the discussions should begin early, but the exact age is still controversial [8]. Some studies have found improvement with later age of transition [13]. In other locations, transition is guided by age irrespective of cognitive development and outcomes vary [14]. The timing of transition is often governed by local structural circumstances such as insurance and clinic age limits instead of the cognitive capacity and ability of the YPLHIV [5].

### Mobility of YPLHIV

Late adolescence and young adulthood are a time of mobility around the world. Some YPLHIV need to be mobile to pursue education in colleges and universities which are often in other regions or localities from where they have been receiving HIV care. Other YPLHIV are following economic opportunities. Mobility can be intricately linked to income generation, family circumstances, climate change, or violence [15]. A recent study in the USA found that YPLHIV below the age of 24 had much higher rates of state-to-state migration than all other ages [16].

Mobility of YPLHIV make transitions to adult care challenging. Economic challenges include changes in insurance coverage from one state to another, lack of access to Ryan White support services in a new state, and financial untethering from parents. Some YPLHIV have insufficient autonomy to navigate establishing HIV care in a new location. This means that they lack capacity, skill, and finances to navigate insurance changes, transportation, scheduling appointments, and managing their HIV care independently. YPLHIV could also be moving to locations that lack HIV services, or these services may be some distance away.

Mobility of YPLHIV can have a direct impact on the transition process between health care teams. Without a concerted effort from the home clinic and the receiving clinic, there is unlikely to be a warm handoff about the client’s HIV-related health care. A warm handoff, where the young person has a chance to meet a member of the adult clinic prior to transition, has been shown to be quite valuable in transition outcomes [17–19].

### Stigma and Disclosure Issues

Many YPLHIV experience various forms of stigma [2]. Stigma is the preconception that one will be treated in a discriminatory way due to either race, gender, sexual identity, or HIV status based upon previous lived experiences [20]. Stigma is a powerful force for YPLHIV and it hinders comfort in clinical and non-clinical settings [21]. Stigma affects all parts of the continuum of care including transition to adult care because it prevents YPLHIV from accessing care at their convenience without hinderance or discrimination [22]. Stigma and discrimination can negatively impact mental health especially during the transition period [5]. Stigma and discrimination can exacerbate depression, anxiety, and substance use all of which can lead to lack of ART adherence during the critical transition period [22].

Self-disclosure of HIV status (becoming aware of your HIV status) is a pivotal point in life for children and young people living with PAH. Typically, disclosure of HIV status to a child is a multistep process over several years that involves close collaboration between the parents and health care team [23, 24]. Despite strong evidence of disclosing HIV status to school age children (6–12 years old), there is still a high degree of variability in the age at which caregivers and parents disclose this information [25, 26]. This reluctance to disclose can be driven by parents, health care providers, and the cognitive capacity of the adolescent. It often takes years for YPLHIV to comprehend the implications of their HIV status [27]. It can be a long adjustment period which can result in both improved adherence or worse adherence [28]. Thus, beginning discussions about transitioning to adult care often feel overwhelming for patients and families immediately after disclosure.

Successive disclosure of one’s HIV status to new people is a powerful force that inhibits transition to adult care. YPLHIV fear clinical spaces such as a dedicated adult HIV clinics because they feel that their HIV status will be inadvertently disclosed to everyone else in the waiting room [27]. YPLHIV also fear disclosure to new health care providers and team members and often meet new team members with skepticism even though they know the team members could help them [27].

### Descriptive Studies

Many descriptive and qualitative studies have addressed the issue of transitioning YPLHIV to adult care and a select sample of these is included in Table 1. These studies query key stakeholders in the transition process including YPLHIV, health care providers, caregivers, and policymakers. Broadly this research elicits barriers and facilitators to transition to adult care.

**Table 1:** Summary of Select Descriptive Studies on the Transition to Adult Care for YPLHIV since 2019

Authors	Title	Publication and Date	Site/Location	Number of Subjects	Ages	Stakeholders Included	Study Type	Outcomes
Ayuk AC, et al. [54]	Pre-Transition Readiness in Adolescents and Young Adults with Four Chronic Medical Conditions in South East Nigeria—An African Perspective to Adolescent Transition	Adolescent Health, Medicine and Therapeutics, 2020	Single Enugu, Nigeria	142	12–24	Patients	Observational (survey)	• All participants had suboptimal transition readiness scores. The older age groups were less willing to transfer to adult care • Children attending the HIV clinic scored highly in independently taking medications, yet medication adherence was not associated with increased likelihood of accepting a move to adult care
Gitahi-Kamau N, et al. [31]	The Role of Self-Efficacy in HIV treatment Adherence and its interaction with psychosocial factors among HIV Positive Adolescents in Transition to Adult Care in Kenya	Vulnerable Child Youth Stud, 2022	Single Kenya	82	16–19	Patients	Observational (survey)	• Expected benefits of ART adherence and self-efficacy are mediated by low self-esteem Interventions aimed at adolescents’ transition to adult care should evaluate individuals’ self-esteem and provide psychosocial services to improve ART adherence
Hussen SA, et al. [7]	Human Immunodeficiency Virus (HIV) Care Continuum Outcomes After Transition to Adult Care Among a Prospective Cohort of Youth with HIV in Atlanta, Georgia	Clinical Infectious Diseases, 2023	Single Atlanta, Georgia, USA	70	Mean age 24	Patients	Observational	• In this HIV care center containing a pediatric and adult-oriented clinic, the initial linkage of youth with HIV to adult care was high, but declined significantly over the 2-year follow-up period, • Additional support may be useful for all adult patients and not just those who recently transitioned out of pediatric care • Viral suppression was maintained in those who remained engaged in care
Isah C, et al. [29]	"The change was too sudden": Experiences of transition to adult care for adolescents living with HIV in North-Central and North-Western Nigeria	International AIDS Society Conference, 2022	Multi Nigeria	149	16–18	Patients	Qualitative	• Adolescents felt uninformed about transition. There was no coordinated transfer to adult care • Adolescents in this Nigerian cohort have gaps in transition preparation, planning and post-transition time periods
Mbalinda SN, et al.^5^ [19]	Transition to adult care: Exploring factors associated with transition readiness among adolescents and young people in adolescent ART clinics in Uganda	PLOS One, 2021	Multi Uganda	786	Mean 17	Patients	Observational (survey)	• Readiness to transition into adult care was significantly associated with having tertiary education, trusting peered educators for HIV treatment, receiving counseling on transition to adult services, and previously visiting an adult clinic to prepare for transition • Overall readiness for transition to adult care was still low (6.5%)
Njuguna I, et al. [32]	What happens at adolescent and young adult HIV clinics? A national survey of models of care, transition and disclosure practices in Kenya	Tropical Medicine & International Health, 2020	Multi Kenya	102 clinics	N/A	Clinics	Observational (survey)	• Common services specific to YPLHIV include dedicated clinic days (with the majority being on weekends), designated clinic spaces, support groups, and HIV literacy meetings • Most clinics tracked disclosure of HIV status, though 40% of clinics discussed disclosure with caregivers or youth a median of 2 years later in practice than stated in clinic guidelines • Median age at transition was 20 years
Tassiopoulos K, et al. [13]	Healthcare Transition Outcomes Among Young Adults With Perinatally Acquired Human Immunodeficiency Virus Infection in the United States	Clinical Infectious Diseases, 2020	Multi USA	455	> 18	Patients	Mixed methods	• Patients who transitioned at age 18 or older had more satisfaction with adult care provider/clinic and higher CD4 counts than participants who transitioned before age 18 • Better healthcare self-management skills and more social support during transition were associated with retention in care

Individual barriers to successful transition include insufficient knowledge and lack of autonomy. A large study in Nigeria emphasized that YPLHIV felt unprepared and lacked knowledge about transition to adult care [29]. Another study of adolescents and young adults in Uganda showed that despite preparation services which included transition counseling, and visiting the adult clinic, perceived readiness to transition among YPLHIV was still quite low at 6.5% [19]. Self-efficacy was a major barrier for YPLHIV [30]. Here self-efficacy means “the extent or strength of one’s belief in one’s own ability to complete tasks and attain goals despite environmental and social barriers” and this is closely tied to self-esteem or confidence [31]. In fact, targeting self-efficacy for YPLHIV may result in improved retention in care after transition [13].

Several facilitators were emphasized in the recent qualitative studies. First and foremost, maintaining youth-friendly services even after transition had a powerful effect on linkage and retention [7, 32]. Here youth-friendly services means that services are “*acceptable* (young people are willing to obtain the health services, *appropriate* (the right health services), and *effective* (the right health services are provided in the right way making a positive impact on young people)” [33]. Linkage to an adult space that is youth friendly is vital and can make all the difference in the world for YPLHIV especially in the beginning when they feel vulnerable [27, 34]. In practical terms, these services make YPLHIV feel like a very important person and could include a welcome tour, a warm handoff, and other specialized and focused care during and after transfer [27, 35]. Other facilitators include mental health support and communication between pediatric and adult care providers [7, 18].

### Interventions for Improving Transitions to Adult Care

Several categories of interventions have been studied to improve outcomes for YPLHIV transitioning to adult care in the last five years (See Table 2). Categories include transition readiness assessments/scales, transition protocol development and education, mobile health interventions, specialized transition clinics, and transition navigation. Many studies included more than one of these categories of intervention and almost all the programs centered on youth-friendly services.

**Table 2:** Summary of Select Intervention Studies on the Transition to Adult Care for YPLHIV since 2019

Authors	Title	Date, Journal/Conference	Site/Location	Number of Subjects	Ages	Study Population (PAH/NPAH/Both)	Study Type	Intervention Description	Results	Implications
Ashaba S, et al. [55]	Development and Validation of the Transition Readiness Assessment Tool (TREAT) for Adolescent and young adults living with HIV in Southwestern Uganda	International Workshop on HIV & Adolescence, 2022	Multi Uganda	300	Mean 19.1	PAH	Intervention with validation	Transition readiness assessment tool (TREAT) was developed and validated	23-item transition readiness scale was internally consistent with good test–retest reliability and validity	This scale is a reliable and valid measure of transition readiness and assesses four key areas: self-management, health care navigation, transition preparation, and stigma
Zanoni BC, et al. [44]	Acceptability, feasibility and preliminary effectiveness of the mHealth intervention, InTSHA, on retention in care and viral suppression among adolescents with HIV in South Africa: a pilot randomized clinical trial	AIDS Care 2024	Multi South Africa	80	15–19	PAH	Randomized clinical trial	Interactive Transition Support for ALHIV (InTSHA) is a mobile health intervention that utilizes group chats to provide peer support and facilitate communication with other ALHIV, caregivers, and healthcare providers during transition	InTSHA intervention group reported high feasibility and acceptability scores. No differences in retention and viral suppression were noted	InTSHA is both a feasible and an acceptable mHealth intervention that needs more long-term study
Brundrett M, et al. [42]	Development, pilot implementation, and preliminary assessment of a transition process for youth living with HIV	J Pediatr Nurse, 2023	Single USA	16	13–24	Both	Pilot Implementation and evaluation	Transition Process for YLHIV will include four stages 1) introduction to transition 2) building knowledge and skills 3) growing in independence 4) adult care ready. Each stage will have competencies that the patient should complete before next stage, and tasks for the care team. Educational materials will be given to patients and caregivers	Most patients felt that the transition protocol’s educational materials were at least moderately helpful. Most felt they were more knowledgeable about their transition after receiving the materials. 3/7 surveyed staff used the accompanying transition protocol	Patients and caregivers found that this intervention is a helpful approach for transition preparation, but it is unclear whether the protocol affects other markers of successful transition, such as viral suppression or retention
Chew et al. [27]	Mixed Methods Evaluation of a Youth-Friendly Clinic for YPLHIV Transitioning from Pediatric Care	Tropical Med Infectious Disease	Single USA	21	18–26	Both	Single Arm with Intervention	Transition Clinic with Youth Friendly services and transition navigation	Successful transition of a small number of patients	The Transition Clinic can help YPLHIV prepare and actuate transition to adult care
Continisio GI, et al. [48]	The Transition of Care from Pediatric to Adult Health-Care Services of Vertically HIV-Infected Adolescents: A Pilot Study	Front Pediatr, 2020	Single Italy	13	14–20	PAH	Prospective study	Transition clinic involves patients having a joint medical visit with pediatric and adult infectious disease physicians every two months. Patients also attended education sessions and individual and group psychology sessions	Results include increased viral suppression, decreased severe psychological distress, and unchanged self-esteem	Collaboration between pediatricians, adult physicians, and psychologists in a transition clinic is an effective approach to improve patient outcomes, clinically and psychologically. Psychological health should be prioritized to improve patients’ quality of life
Fomo MF, et al. [45	A qualitative assessment of the perceived acceptability and feasibility of eHARTS, a mobile application for transition readiness assessment for adolescents living with HIV in South Africa	PLOS Digital Health, 2023	Multi South Africa	15 patients 15 HCPs	Mean 16.2	PAH	Qualitative	The electronic HIV Adolescents Readiness Transition Scale (eHARTS) is a mobile application that includes a questionnaire and scoring system to predict the patient’s likelihood of viral suppression after transition. The application alerts HCPs of transition readiness category and identifies possible interventions	eHARTS was found to be feasible, acceptable, and helpful by patients and HCPs	This eHARTS mobile health intervention is useful to assess transition readiness and help identify gaps that HCP team can target and prepare adolescents for the next steps
Gitahi N, et al. [41]	Life Skills Improve Psychosocial and Clinical Outcomes Among Adolescents Living with HIV Transitioning to Adult Care	Journal of Adolescent Health, 2023	Single Nairobi, Kenya	82	Not stated	PAH	Randomized controlled trial, mixed methods	Curriculum involves programming designed to build patients’ life skills	Life skills curriculum group had higher ART adherence self-efficacy, viral suppression, and self-esteem than group receiving standard care	Life skills curriculum can address psychosocial needs among YPLHIV and improve adherence, self-efficacy, and self-esteem
Jones SC, et al. [36]	HIV Health Care Transition Readiness: Embracing the Opportunity and Challenge	J Assoc Nurses AIDS Care, 2019	Single USA	48	22–24	Both	Retrospective study with intervention	TRAQ scores were retrospectively assessed among 48 patients with HIV	Use of TRAQ score affected provider interventions for medication management TRAQ did not increase clinical interventions	TRAQ did not necessarily increase clinical interventions, but it can enable providers to plan management tailored to needs of individual clients
Njuguna IN, et al.^2^ [26]	Transition to independent care for youth living with HIV: a cluster randomized clinical trial	Lancet HIV, 2022	Multi Kenya	1066	15–24	Not stated	Cluster randomized clinical trial	Adolescent Transition Package includes tools to support HIV disclosure, guide and track transition discussions, and assess transition readiness	ATP group had higher overall transition readiness and HIV literacy domain scores There were no improvements in viral suppression or retention in intervention group	ATP delivered by health care workers showed effectiveness in improving transition readiness and HIV literacy
Ryscavage P, et al. [39]	Stepping up: retention in HIV care within an integrated health care transition program	AIDS Care, 2022	Single USA	108	Not stated	Both	Retrospective study with intervention	Structured Transition Empowerment Program integrates the adult HIV clinical team into the pediatric and adolescent clinic to develop individualized transition plans and facilitate transfer of care. The protocol outlines which staff members should be present at each stage of transition	94% of STEP program participants were linked to adult care. 12-month retention in the STEP cohort was significantly higher than that of the pre-STEP cohort	Retention in adult care was higher among patients who participated in this integrated pediatric and adult health care transition program
Tanner A, et al. [56]	Developing and Pilot Testing iTransition: A multilevel mHealth Intervention to Support Transition to Adult Care for Youth Living with HIV	Journal of Adolescent Health, 2023	Multi USA	Open enrollment but pursuing 20 control and 50 intervention participants	Not stated	Both	Phase 1 entailed development and usability testing of mHealth intervention, while current Phase 2 is the pilot implementation trial to assess its feasibility, acceptability, and preliminary efficacy	ITransition is a mobile app with educational tools, medication reminders, and readiness assessments for patients, and educational content and platforms to track youth progress for providers	Qualitative interviews with YPLHIV and providers revealed that iTransition had beneficial features, such as medication adherence reminders, communication with providers, and HIV-related resources. However, usage levels were low for youth and providers, as it lacked integration into the electronic health records (EHR) and competed with their other priorities	While participants felt iTransition was potentially useful in improving HIV care continuum outcomes, developers need to first improve its implementation and integration into EHR before it can be feasibly used by both YPLHIV and providers
Vargas V, et al.^3^ [49]	Community Based accompaniment for adolescents transitioning to adult HIV care in urban Peru	AIDS Behavior, 2022	Single Peru	30	18–20	Both	Pilot study to assess feasibility	“PASEO” is a nine-month program offering services such as accompaniment to adult clinic, routine check-in visits, directly observed treatment, social support, educational workshops, and mental health screenings	PASEO improved transition readiness, medication adherence, and psychosocial outcome scores. Most patients who were referred to PASEO participated, and most attended at least one social support group	Transition navigation through PASEO is an intervention that improved transition outcomes
Zanoni B, et al. [57]	Development of a transition readiness score for adolescents living with perinatally-acquired HIV and transitioning to adult care	AIDS Behav, 2022	Single South Africa	199	12–21	PAH	Prospective Analysis	Transition readiness score is calculated based on risk factors associated with viral suppression: age at ART initiation, ART regimen, biological sex, HIV disclosure status, drug/alcohol use, and transition readiness using the HIV Adolescents Readiness Transition Scale (HARTS)	Scores ranged between -8 and 11 and were divided into high, intermediate, and low transition readiness quartiles. A score of 5 and up implied that adolescents would have a high likelihood of viral suppression without additional intervention, whereas scores below 5 would benefit from additional time in pediatric care	This transition readiness score for adolescents with PAH can identify who is ready to transition to adult care and who may first need additional resources for modifiable factors like disclosure and drug/alcohol use

Implementation of transition readiness assessments or scales has been a widespread intervention that has shown validity and effectiveness. Jones et al. utilized the validated Transition Readiness Assessment Questionnaire (TRAQ) to longitudinally follow YPLHIV as they prepare to transition to adult care [36]. Health care team members provided interventions and support to target skill deficits, and the team was able to follow each person’s progress using the TRAQ. Another group developed and validated a transition readiness scale called HIV Adolescent Readiness to Transition Scale (HARTS) utilized in South Africa which had a strong correlation to successful transition to adult care [37]. The HARTS scores allowed providers to target YPLHIV who needed more resources and education to successfully complete transition. It is important to recognize that many of the validated transition scales focus on gaps in skills or knowledge. Health care teams also need to focus on strengths and resilience factors for YPLHIV as they transition to adult care, which is why tools such as the Social Provision Scale are utilized [38]. The most important use of these tools is to allow medical teams to understand individual stressors and resilience factors during the transition process. This allows tailored support services based on individual factors such as family support, peer support, and educational and vocational goals.

Designing and implementing a formal **transition protocol or process** can be a useful intervention to aid the transfer to adult care for YPLHIV. One of the best examples of this is the Structured Transition Empowerment Program (STEP) which incorporates the six core elements from GOT transition: a transition protocol; an updated registry of pre and post transition retention; a transition readiness assessment; an individualized transition plan; a warm handoff within the STEP Clinic; and a formal transition to the adult HIV care clinic [39, 40]. This program showed remarkably high retention in care after transition to adult care. Another group implemented an Adolescent Transition Package (ATP) which included readiness assessment surveys, disclosure interventions, and formalized education about transition [26]. The YPLHIV who were included in the ATP group had higher transition readiness and HIV literacy compared to controls. Many transition programs include client education on transition skills and self-efficacy [31]. Examples of this include providing educational curriculums for providers and patients and assessing periodically for skills acquisition [41, 42].

**Mobile health interventions** offer a promising future opportunity to help YPLHIV transition to adult care. The Interactive Transition Support for Adolescents Living with HIV using Social Media (InTSHA) is a multi-site study in South Africa that utilizes group chats, peer support, and consistent team communication during transition to adult care [43]. Preliminary results show feasibility and acceptability among YPLHIV; however, impact on successful transition is still being studied [44]. In another multi-site study in South Africa Fomo, et al. found feasibility and acceptability of eHARTS which is a mobile phone-based transition readiness assessment tool for YPLHIV [45]. Tanner et al. are currently studying iTransition which is a mobile application that provides education, adherence reminders, and transition progress tracking [46]. This multi-site double-arm observational study in the USA should have results within the next few years. Health care teams who utilize mobile technology need to be keenly aware of using thoughtful language during communication and avoid using stigmatizing language such as HIV, AIDS, or other terms which may inadvertently disclose a person’s status to a third party. Although mobile health interventions have yet to show efficacy with transition outcomes, they represent a unique and promising way to engage YPLHIV during the transition process.

**Transition clinics** have implemented integrated care models to prepare YPLHIV to transition to adult care. Integrated care models incorporate HIV care with other health care services such as contraception or mental health [47]. In one study, researchers implemented an integrated transition clinic with psychological support, transition education, and co-location of pediatric and adult infectious disease experts [48]. In this small single arm study, both viral suppression and psychological distress were improved; however, no measurement of transition outcomes was ascertained. In another small study, Chew et al. showed benefits to viral suppression and successful transition outcomes for a small group of YPLHIV enrolled in a transition clinic [27]. This transition clinic included mental health support, transition readiness education, case management, and a transition navigator. Feedback from clients showed that the transition clinic helped with developing autonomy and that a warm handoff between pediatric and adult providers was a useful intervention for facilitating transition [27].

**Personalized navigation** throughout the transition process may also offer an opportunity for YPLHIV moving to adult care. The best example of this is the “PASEO” program in Peru which utilized a navigator who accompanied YPLHIV to the first few adult clinic visits [49]. The “PASEO” intervention improved transition readiness, medication adherence, and psychosocial outcomes for YPLHIV. Another program utilized a health care transition navigator who successfully engaged YPLHIV through barriers to transition including appointment reminders, transportation resources, insurance navigation, and mental health resources [50]. Some of the most successful transition programs utilize a peer navigator who is typically an individual who has successfully completed the process of transition [5, 34]. Enhanced case management throughout the transition process proved to be valuable for YPLHIV who have numerous social determinants of health [18]. Peer navigation and enhanced case management require infrastructure, funding, and commitment but when they are utilized with other comprehensive interventions, they have tremendous efficacy.

## Recommendations

Several recommendations can be suggested based on the qualitative and interventional studies for teams working on transitioning YPLHIV to adult care.

- Begin communicating with the patient and family about the process of transition at an early age but allow enough time after self-disclosure [25].
- Utilize transition readiness assessments and tailor support to individuals who need more resources prior to and after transition [8, 37].
- Communication between pediatric and adult care providers during the transfer process is vital and a warm handoff is effective [17, 18].
- Mobile communication through text, applications, or health portals offers an opportunity to engage YPLHIV throughout the transition process [45, 46].
- Adult HIV care clinics can adopt youth-friendly services to engage YPLHIV as they transfer into care [12, 51].
- Transition interventions need to be anchored to the local and regional community [5].
- YPLHIV should be involved in program development and research about transition to adult care [27, 52, 53].

## Conclusion

Transitioning YPLHIV to adult care is a complex but a good problem to have. Simple interventions such as developing a transition protocol, implementing transition assessments, and training adult clinics in youth-friendly services can have a major impact on the process. When possible good communication between pediatric and adult providers should also be utilized. Indeed, transition to adult care for YPLHIV is an opportunity for both pediatric and adult HIV care teams. We all have a vested interest in maintaining a patient’s viral suppression and emotional and psychological wellbeing during young adulthood.

## Key References

- Dahourou, D.L., et al., *Transition from paediatric to adult care of adolescents living with HIV in sub-Saharan Africa: challenges, youth-friendly models, and outcomes.* J Int AIDS Soc, 2017. **20**(Suppl 3): p. 21,528. 
  - This narrative review summarizes the studies aimed at transition to adult care for YPLHIV in a region with the highest case load.
- Momplaisir, F., et al., *Strategies to improve outcomes of youth experiencing healthcare transition from pediatric to adult HIV care in a large U.S. city.* Arch Public Health, 2023. **81**(1): p. 49.
  - This qualitative study ellicits experiences from clinicians and YPLHIV about the transition process.
- Society for Adolescent, H. and Medicine, *Improving Outcomes for Adolescents and Young Adults Living With HIV.* J Adolesc Health, 2023. **73**(3): p. 605–609. 
  - This position paper from the Society for Adolescent Health and Medicine reviews effective interventions for improving the continuum of care for YPLHIV.

## Data Availability

No datasets were generated or analysed during the current study.
